# A Laminin G-EGF-Laminin G Module in Neurexin IV Is Essential for the Apico-Lateral Localization of Contactin and Organization of Septate Junctions

**DOI:** 10.1371/journal.pone.0025926

**Published:** 2011-10-14

**Authors:** Swati Banerjee, Raehum Paik, Rosa E. Mino, Kevin Blauth, Elizabeth S. Fisher, Victoria J. Madden, Alan S. Fanning, Manzoor A. Bhat

**Affiliations:** 1 Department of Cell and Molecular Physiology, University of North Carolina School of Medicine, Chapel Hill, North Carolina, United States of America; 2 Curriculum in Neurobiology, University of North Carolina School of Medicine, Chapel Hill, North Carolina, United States of America; 3 Microscopy Services Laboratory, Department of Pathology and Laboratory Medicine, University of North Carolina School of Medicine, Chapel Hill, North Carolina, United States of America; 4 UNC-Neuroscience Center, University of North Carolina School of Medicine, Chapel Hill, North Carolina, United States of America; 5 Neurodevelopmental Disorders Research Center, University of North Carolina School of Medicine, Chapel Hill, North Carolina, United States of America; National Institutes of Health (NIH), United States of America

## Abstract

Septate junctions (SJs) display a unique ultrastructural morphology with ladder-like electron densities that are conserved through evolution. Genetic and molecular analyses have identified a highly conserved core complex of SJ proteins consisting of three cell adhesion molecules Neurexin IV, Contactin, and Neuroglian, which interact with the cytoskeletal FERM domain protein Coracle. How these individual proteins interact to form the septal arrays that create the paracellular barrier is poorly understood. Here, we show that point mutations that map to specific domains of *neurexin IV* lead to formation of fewer septae and disorganization of SJs. Consistent with these observations, our *in vivo* domain deletion analyses identified the first Laminin G-EGF-Laminin G module in the extracellular region of Neurexin IV as necessary for the localization of and association with Contactin. Neurexin IV protein that is devoid of its cytoplasmic region is able to create septae, but fails to form a full complement of SJs. These data provide the first *in vivo* evidence that specific domains in Neurexin IV are required for protein-protein interactions and organization of SJs. Given the molecular conservation of SJ proteins across species, our studies may provide insights into how vertebrate axo-glial SJs are organized in myelinated axons.

## Introduction

In *Drosophila,* the pleated septate junctions (SJs) form a barrier to the paracellular movement of ions and macromolecules that is conserved across species [1], [2], [3]. The epithelial SJs in *Drosophila* are present at the apico-lateral surface of the cells below the adherens junctions (AJs) and have a signature ladder-like morphology [4], [5], [6]. SJs are important for epithelial morphogenesis in invertebrates, and they also provide a paracellular diffusion barrier [2], [7], [8] that maintains ionic environment required for nerve impulse conduction. In addition, they support signaling events that regulate cell division and differentiation [9]. Invertebrate SJs share similarity in structure and molecular composition with the vertebrate paranodal axo-glial SJs that form a molecular barrier critical for the maintenance of axonal domains in myelinated nerves [6], [10], [11].

*Drosophila* SJs and vertebrate axo-glial SJs consist of a core complex of three cell adhesion molecules. These are *Drosophila* Neurexin IV (Nrx IV), Contactin (Cont) and Neuroglian (Nrg) and their vertebrate orthologs Caspr, Contactin and Neurofascin 155 [8], [10], [12], [13]. These three proteins are interdependent for their SJ localization [8], [13], and loss of any one of these proteins in either *Drosophila* or mouse disrupts SJs and the characteristic barrier function [2], [13], [14], [15], [16]. The cytoplasmic domain of *Drosophila* Nrx IV interacts with the cytosolic FERM domain protein Coracle (Cora), while the vertebrate homolog Caspr interacts with Cora homolog Band 4.1 [17], [18]. Numerous other SJ proteins have been identified that are required for SJ formation. These include the MAGUK proteins Discs large [19], [20] and Varicose [21], [22], the claudin-related proteins Sinuous [23], Megatrachea [24] and Kune-kune [25], the Na K-ATPase [26], the cell adhesion protein Lachesin [27] and a more recently discovered Ly6 family of GPI anchored proteins Boudin [28], Crooked, Crimpled and Coiled [29]. How these proteins are involved in the assembly of SJs is still not fully understood.

Structure/function analyses have provided key insights into protein domain(s) in cellular contexts as diverse as morphogenesis [20], [30], signal transduction [31], photoreceptor organization [32] and protein trafficking [33], [34], [35]. While a wealth of information has emerged on the multi-functional roles of *Drosophila* Nrx IV since its discovery [12], [36], [37], no structure/function analyses have been reported thus far that provide insights into the mechanistic role of Nrx IV in SJ organization. Here we report the first structure-function analysis of *Drosophila* Nrx IV in epithelial SJ organization *in vivo.* Biochemical and ultrastructural analyses of *nrx IV* hypomorphic alleles and *nrx IV* null mutants expressing Nrx IV transgenes with domain-specific deletions reveal that the first Laminin G-EGF-Laminin G (LEL1) unit from the extracellular domain (ECD) of Nrx IV is essential for binding to Cont and SJ organization. Reintroduction of the LEL1 unit alone is sufficient for targeting of both Nrx IV and Cont to the apico-lateral domain of the plasma membrane and retains its ability to associate in a molecular complex with Cont but is not able to restore a full complement of SJs between the epithelial membranes. Together our studies reveal that the assembly and function of invertebrate SJs requires coordinated interactions between both membrane-associated and cytoskeletal proteins.

## Materials and Methods

### *Drosophila* Stocks

*Canton S* was used as wild type control. Mutant strains that have been previously described are: *nrx IV^4304^, nrx IV^1817^, nrx IV^319^, nrx IV^711^* and *nrx IV^2511^* [12]. The following reagents were generated for this study: *UAS-nrx IV^myc^, UAS-nrx IV^mycΔDL^, UAS-nrx IV^mycΔLEL1^, UAS-nrx IV^mycΔLEL2^, UAS-nrx IV^mycΔDL-LEL1^, UAS-nrx IV^mycΔLEL1-LEL2^, UAS-nrx IV^mycΔNT^*, and *UAS-nrx IV^mycΔCT^* and *UAS-nrx IV^mycLEL1^*. *arm-Gal4* [38] and *Act5C-Gal4* were obtained from the Bloomington Stock Center.

### Generation of myc-tagged Nrx IV and its Mutant Forms

To generate a 6xmyc-tagged Nrx IV (Nrx IV^myc^), an Rsr II restriction site was created in *nrx IV* cDNA at the coding sequence nucleotide number 3590 using primer sets shown below. The PCR fragments and the vector [*pBS-SK(+)*] were cloned in a three-way ligation (Not I-Rsr II + Rsr II-Xba I + Not I-Xba I). After confirming the presence of Rsr II restriction site in *nrx IV* cDNA, a PCR fragment flanked by Rsr II restriction sites encoding a 6xMyc epitope tag was generated using primers shown below. The amplified fragment was inserted into the newly created Rsr II site in *nrx IV* cDNA. The *nrx IV^myc^* cDNA was subsequently cloned into Not I and Xba I of *pUAST* vector to generate transgenic flies. The expression of Nrx IV^myc^ protein was confirmed both by immunoblotting and immunostaining using anti-Nrx IV and anti-Myc antibodies. To generate mutant forms of *nrx IV^myc^* that lacked specific domains, the external 5′ and 3′ primers were the same as in the full-length cDNA. The internal primers used for deletions were flanked by Nde I restriction sites. For expression in transgenic flies cDNAs encoding *nrx IV^myc^* and various mutant versions were all cloned into *pUAST* except *nrx IV^mycLEL1^* which was cloned into *pUAST-attB*.

Following primers were used for generating *nrx IV^myc^* and various truncations

*nrx IV* containing RsrII site

(Not I) 5′-CCTGGAGCGGCCGCCTCTTCCGAAGTGGGCGTGATC-3′

(Rsr II) 5′-CCAAGGGTGGTGGCCTCGGTCCGAGTCTCGATTTCAATGGGC-3′

(Rsr II) 5′-GCCCATTGAAATCGAGACTCGGACCGAGGCCACCACCCTTGG-3′

(Xba I) 5′-CCTGGATCTAGATTAGATAAAGATCTCTGTTCGCTTCC-3′

*nrx IV^myc^*

(Rsr II) 5′-CCCGGACCGGTACCCGGGGATCCCATCGA-3′

(Rsr II) 5′-CCCGGTCCGAACATCTCGAGAGGCCTTGA-3′

*nrx IV^mycΔDL^*

(Nde I) 5′-CCTGGACATATGTTCCATCAGCGGCTGGTTGCAATC-3′

(Nde I) 5′-CCTGGACATATGAACACGATCTACGCGTGTCCTTCG-3′

*nrx IV^mycΔLEL1^*

5′-CCTGGACATATGCTCTCCCAGTTCAGTGCTGTCTTTC-3′

5′-CCTGGACATATGGTGACCTTCCGCATTGCCGATGCT-3′

*nrx IV^mycΔLEL2^*

5′-CCTGGACATATGGAACAGATCATCGCCCTCACAGCG-3′

5′-CCTGGACATATGCTGATGTTCCAGCAGAATCCTCCC-3′

*nrx IV^mycΔDL-LEL1^*

5′-CCTGGACATATGTTCCATCAGCGGCTGGTTGCAATC-3′

5′-CCTGGACATATGGTGACCTTCCGCATTGCCGATGCT-3′

*nrx IV^mycΔLEL1-LEL2^*

5′-CCTGGACATATGCTCTCCCAGTTCAGTGCTGTCTTTC-3′

5′-CCTGGACATATGCTGATGTTCCAGCAGAATCCTCCC-3′

*nrx IV^mycΔNT^*

5′-CCTGGACATATGTTCCATCAGCGGCTGGTTGCAATC-3′

5′-CCTGGACATATGCTGATGTTCCAGCAGAATCCTCCC-3′

*nrx IV^mycLEL1^*

5′-CCTGGACATATGAACACGATCTACGCGTGTCCTTCG-3′

5′-CCTGGACATATGGAACAGATCATCGCCCTCACAGCG-3

*nrx IV^mycΔCT^*

(Not I) 5′-CCTGGAGCGGCCGCCTCTTCCGAAGTGGGCGTGATC-3′

(Xba I) 5′-GTCGCCTTCTAGATTAGCGACCGCGACCGATAAGGAAGAACATAAGG-3′

### Immunostaining of Embryos

Immunostaining of stage 16 embryos was carried out as previously described [13]. Primary antibodies used were: rabbit anti-Nrx IV (1∶500) [12], guinea pig anti-Cont (1∶1500) [8], and anti-Myc (1∶1000, Cell Signaling). Isotype specific and fluorescent secondary antibodies Alexa 488, 568 and 647 were obtained from Jackson Immunochemicals and Invitrogen, respectively. Immunofluorescence images of embryos were captured under identical settings with a Z-step of 0.3 µ on a BioRad Radiance 2000 confocal microscope and processed with Adobe PhotoShop software.

### Isolation of Homozygous Mutant Embryos

*nrx IV^1817^, nrx IV^319^, nrx IV^711^* and *nrx IV^2511^* mutants were balanced with *twi-GFP* balancer chromosomes and respective homozygous mutant (non-GFP) embryos were staged and sorted using a Leica MZ16FA fluorescence stereomicroscope. Wild-type *Canton S* embryos were identically processed and used as controls.

### Immunoprecipitation and Immunoblotting

Embryos of desired genotypes were homogenized using a glass homogenizer in a weight/volume ratio of 1∶5 in ice cold lysis buffer containing 50 mM HEPES (pH 7.2), 100 mM NaCl, 1 mM MgCl_2_, 1 mM CaCl_2_ and 1% NP-40 with protease inhibitors. The lysates were kept on ice for 20 minutes and centrifuged at 15,000× g for 30 minutes at 4°C followed by re-centrifugation, and used subsequently for immunoprecipitation (IP). For each IP reaction, 100 µl of supernatant was precleared with Protein A beads followed by incubation with primary antibodies at 1∶100 dilution (anti-Cont) for 8 hours at 4°C. The supernatant-antibody mix was incubated with 25 µl of pre-washed Protein A beads for 2 hours at 4°C. The beads were then washed three times in PBS followed by elution of the immunocomplexes in 30 µl of PBS/SDS buffer and resolved on SDS-PAGE for immunoblotting with respective antibodies. All IP experiments were carried out using identical experimental conditions. Dilutions of primary antibodies used for western blot were: Mouse anti-Myc (1∶50,000); rabbit anti-Nrx IV (1∶1500); guinea pig anti-Cont (1∶1500) and mouse anti-β-Tubulin (1∶10,000).

### Transmission Electron Microscopy (TEM)

*nrx IV^1817^, nrx IV^2511^, nrx IV^711^, nrx IV^319^,arm-Gal4/UAS-nrxIV^myc^;nrx IV^4304^/nrx IV^4304^, arm-Gal4/UAS-nrxIV^mycΔDL^;nrx IV^4304^/nrx IV^4304^, arm-Gal4/UAS-nrxIV^mycΔLEL1^;nrx IV^4304^/nrx IV^4304^, arm-Gal4/UAS-nrxIV^mycΔLEL2^;nrx IV^4304^/nrx IV^4304^, arm-Gal4/UAS-nrxIV^mycΔDL.LEL1^;nrx IV^4304^/nrx IV^4304^, arm-Gal4/UAS-nrxIV^mycΔLEL1.LEL2^;nrx IV^4304^/nrx IV^4304^, arm-Gal4/UAS-nrxIV^mycΔNT^;nrx IV^4304^/nrx IV^4304^, arm-Gal4/UAS-nrx IV ^mycΔCT^;nrx IV^4304^/nrx IV^4304^ and arm-Gal4/UAS-nrx IV^mycLEL1^;nrx IV^4304^/nrx IV^4304^* mutant embryos were identified against a *twi-GFP* balancer chromosome. Mutant and wild type embryos of 19-21 hours were processed for epithelial TEM analysis as described previously [4], [13].

### Dye Exclusion Assay

Control (*nrx IV^4304^/TM3, twi-GFP*) and mutant (arm-Gal4/UAS-nrx IV^myc*^;nrx IV^4304^/nrx IV^4304^) stage 16 embryos (n = 15) were collected and processed for dye injections as previously described [13]. Rhodamine (3 mg/ml)-labeled dextran (10,000 molecular weight, neutral; Invitrogen) in injection buffer (5 mM KCl, 0.1 mM sodium phosphate, pH-6.8) was injected into the body cavity at the posterior end of the embryo using an Eppendorf micromanipulator FemtoJet. The embryos were visualized under a Zeiss LSM 510 confocal microscope and imaged simultaneously 15 minutes after injection.

### Statistics

Statistical analysis on 15 embryos of the various genotypes were performed using ANOVA followed by Tukey range test and error bars indicate SEM. * indicates a *p* value of <0.05, ** indicates a *p* value of <0.01, and *** indicates a *p* value of <0.001.

## Results

### Hypomorphic Alleles of *nrx IV* Fail to Organize a Full Complement of SJs

**Figure 1:**
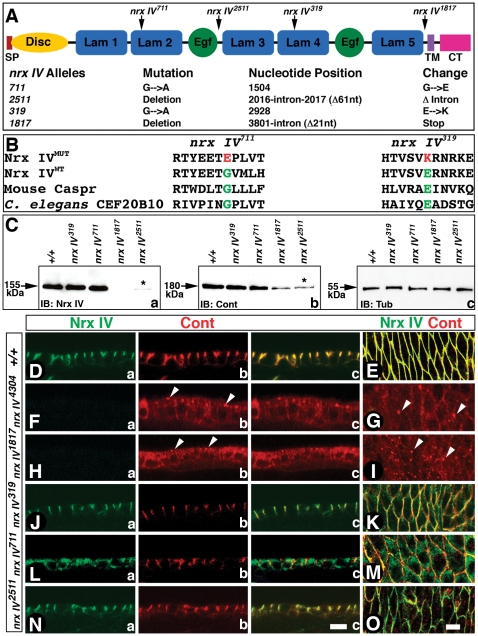
Domain-specific *nrx IV* mutant alleles show altered localization of Cont. (**A**) Schematic of the primary structure of Nrx IV shows the signal peptide (SP), a Discoidin (Disc) domain, 5 Laminin G (Lam 1-Lam 5)) domains, 2 epidermal growth factor-like repeats (EGF), transmembrane (TM) and the cytoplasmic (CT) region. Genomic sequence analysis revealed nucleotide changes in specific *nrx IV* alleles. Mutations in various *nrx IV* alleles that were identified by sequence analysis are shown above the protein domains. The sequence numbers refer to the nucleotide sequence of the *nrx IV* cDNA [12]. (**B**) The amino acid changes in *nrx IV^711^* and *nrx IV^319^* occur at highly conserved amino acids in laminin G domains. In *nrx IV^711^* allele a highly conserved glycine in the second laminin G domain has changed to glutamic acid and in the *nrx IV^319^* allele a highly conserved glutamic acid has changed to lysine. Note that these amino acids are conserved from *C. elegans* to vertebrates [39]. (**C**) Western blot analysis shows Nrx IV (**a**), Cont (**b**) and Tub (**c**) levels in wild type and various *nrx IV* homozygous mutants. (**D, E**) Wild type embryonic epithelia show Nrx IV (**a**) and Cont (**b**) colocalization (**c**) at the SJs. Surface view of the epithelial plasma membrane show colocalization of Nrx IV and Cont (**E**). (**F–I**) *nrx IV^4304^* (**F, G**) *and nrx IV^1817^* (**H, I**) embryos show absence of Nrx IV (**Fa, Ha**) and diffuse and punctate Cont distribution in the epithelia (**Fb, c , G, Hb,c, I**). (**J, K**) *nrx IV ^319^* embryos show Nrx IV localization at the SJs (**Ja**) and some expression in the cytoplasm (**K**). Cont localization seems unaffected at the SJs (**Jb**). Cont colocalizes with Nrx IV in the epithelial membrane (**K**). (**L, M**) *nrx IV^711^* embryos show a more basolateral distribution of Nrx IV and a higher accumulation in the cytoplasm (**La, c**). Cont shows membrane localization and in some cells enrichment at the epithelial SJs (**Lb, c**). Surface view shows Nrx IV and Cont colocalization in membrane and additional Nrx IV localization in cytoplasm (**M**). (**N, O**) *nrx IV^2511^* embryos show severely reduced Nrx IV at SJs (**Na, c; O**) and Cont localization in also reduced in the epithelial membrane and SJs (**Nb, c, O**). This severe decrease is most apparent in enface views. Scale bars: 10 µm.

The primary structure of Nrx IV is characterized by the presence of a signal peptide, a discoidin (Disc) domain, a laminin (Lam) G domain and two modules of laminin G-EGF-laminin G (LEL) in its extracellular region followed by a transmembrane (TM) and a short 48 amino acid cytoplasmic (CT) region (Fig. 1A) [12]. To determine the domain requirements of Nrx IV for proper Cont localization and epithelial SJ organization, we characterized previously isolated *nrx IV* EMS alleles: *nrx IV^1817^, nrx IV^319^, nrx IV^711^* and *nrx IV^2511^* (Fig. 1), in addition to the more extensively studied *nrx IV^4304^* null allele [8], [12], [13], [14], [36]. The mutations in these alleles are summarized in the schematic (Fig. 1A). *nrx IV^319^* and *nrx IV^711^* alleles carry missense mutations in two different laminin G domains (Fig. 1A, B). The affected residues in these alleles are highly conserved between the Neurexin IV/Caspr/Paranodin (NCP) family members, suggesting an important functional role in Nrx IV function (see below) (Fig. 1B) [39], [40]. *nrx IV^1817^* showed a deletion of 16 nucleotides including nucleotides 3797–3801 of an exon, as well as 11 nucleotides from the next intron. This resulted in a frame shift adding an additional 9 unrelated amino acids. *nrx IV^2511^* had a 61 nucleotide deletion in an intron (2016-intron-2017) (Fig. 1A). *nrx IV^711^* and *nrx IV^319^* alleles have amino acid changes in highly conserved regions of the second and fourth laminin G domains, respectively (Fig. 1B) [39].

To determine whether *nrx IV* hypomorphic mutations affected Nrx IV protein levels in embryos, we carried out immunoblot analysis of the homozygous wild type (*+/+*), *nrx IV^319^, nrx IV^711^*, *nrx IV^1817^* and *nrx IV^2511^* using anti-Nrx IV antibodies raised against the C-terminus of Nrx IV [12]. This revealed near normal levels of Nrx IV in *nrx IV^319^* and *nrx IV^711^* embryos when compared to wild type (Fig. 1Ca). *nrx IV^1817^* showed absence of Nrx IV indicating that this is a protein null allele. Interestingly, *nrx IV^2511^* homozygous embryos showed significantly reduced Nrx IV (Fig. 1Ca, asterisk) and is classified as a hypomorphic allele. Since Nrx IV is known to interact with Cont and is required for its SJ localization [8], we tested whether Cont levels were affected in *nrx IV* alleles. Immunoblot analysis using anti-Cont antibodies showed that Cont levels were relatively unaffected in *nrx IV^319^* and *nrx IV^711^* alleles (Fig. 1Cb) but were severely reduced in *nrx IV^1817^* and *nrx IV^2511^* alleles (Fig. 1Cb, asterisk). Immunoblots using anti-β-Tubulin antibodies showed that equal amount of protein was present in all the lanes (Fig. 1Cc).

We next determined the localization pattern of Nrx IV and Cont in the epithelia of stage 16 *nrx IV* hypomorphic mutants described above. Both Nrx IV (Fig. 1Da) and Cont (Fig. 1Db) colocalize at the plasma membrane and are apically enriched at the SJs in the wild type epithelia (Fig. 1Dc; E). *nrx IV^4304^* and *nrx IV^1817^* have no detectable Nrx IV (Fig. 1Fa, c, G; Ha, c, I) and a diffuse and punctate Cont localization in the embryonic epithelia (Fig. 1Fb, c, G; Hb, c, I, arrowheads). In *nrx IV^319^* embryos, which have near wild type levels of Nrx IV, both Nrx IV and Cont still concentrate in apical SJ area (Fig. 1Ja–c, K), although Nrx IV also showed an increase in cytoplasmic localization relative to wild type embryos (Fig. 1Jc, K). *nrx IV^711^* displayed a higher level of Nrx IV accumulation in the cytoplasm and a mislocalization along the more basolateral domains of the epithelial membrane, while Cont did not show normal apical-lateral localization (Fig. 1La–c, M). Interestingly, *nrx IV^2511^* showed significantly reduced levels of Nrx IV (Fig. 1Na, c; O) and Cont (Fig. 1Nb, c; O) at the SJs. This is particularly evident from enface section (Fig. 1O). These data demonstrate that specific mutations in Nrx IV alter its localization as well as that of Cont at the SJs and that reduction in Nrx IV protein levels also affects the accumulation of Cont at epithelial SJs.

**Figure 2:**
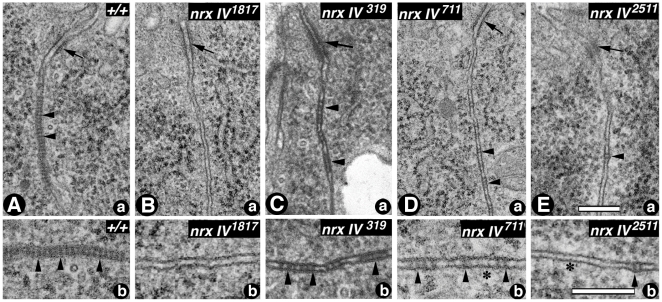
*nrx IV* mutant alleles display defective organization of epithelial SJs. (**A**) TEM of the wild type embryonic epithelia (+/+) shows apical AJs (**a**, arrow) basal to which is the compact band of SJs (**a**, arrowheads). A higher resolution image of the SJs (**Ab**, arrowheads). (**B**) *nrx IV^1817^* embryos show complete loss of SJs (**a, b**) between the epithelial membranes below the AJs (**a**, arrow). (**C**) *nrx IV^319^* embryos show properly formed septa (**a, b**, arrowheads). The SJs are formed in the apical lateral region of the epithelial cells. (**D**) *nrx IV^711^* embryos show sparsely formed septa (**a, b**, arrowheads) with clear gaps in between the septa (**b**, asterisk). The SJs extend further along the basolateral area of the epithelia (a, arrowheads). (**E**) *nrx IV^2511^* embryos show presence of very few septa (**a, b**, arrowhead) between the epithelial membranes. Scale bars: Aa-Ea, 0.2 µm; Ab-Eb, 0.1 µm.

Given the consequence of *nrx IV* hypomorphic and null mutations on Cont localization and stability at epithelial SJs, we wanted to determine whether these changes translate into ultrastructural defects in the organization of SJs. We carried out TEM of wild type (Fig. 2A) and the mutant *nrx IV* alleles: *nrx IV^1817^*, *nrx IV^319^*, *nrx IV^711^* and *nrx IV^2511^* (Fig. 2B–E, respectively). Wild type embryonic epithelia showed the presence of apical AJs (Fig. 2A, arrow) below which are the characteristic ladder-like pleated SJs (Fig. 2Aa, b, arrowheads). *nrx IV^1817^* showed a complete lack of SJs (Fig. 2Ba, b) but had normal AJs (Fig. 2Ba, b, arrow). *nrx IV^319^*, which has a mutation in the LEL2 module of Nrx IV showed presence of SJs characterized by discontinuous clusters of septae (Fig. 2Ca, b, arrowheads). *nrx IV^711^*, with the mutation in LEL1, formed small isolated septae that were along the basolateral region of the epithelia which is not observed in the wild type epithelia (Fig. 2Da, b, arrowheads, compare with Fig. 2Aa). The other LEL1 mutant *nrx IV^2511^* (Fig. 2Ea, b, arrowheads) had even fewer septae along the basal lateral membrane than *nrx IV^711^* (Fig. 2D) but also revealed normal AJs (Fig. 2Ea, b, arrows). These data demonstrate that mutations in LEL1 domains of Nrx IV and reduced expression of Nrx IV affect the formation of SJs, thus highlighting the functions of individual domains in the organization of SJs.

### Generation of Domain-Specific Deletions in Nrx IV and Their *in vivo* Expression

**Figure 3:**
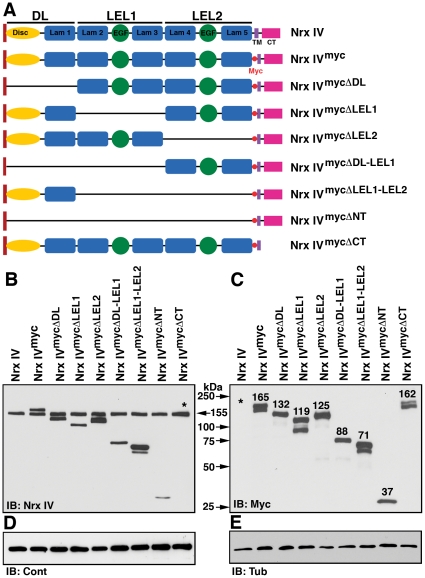
*In vivo* Expression of domain-specific deletion forms of Nrx IV. (**A**) Schematics of the wild type Nrx IV with the extracellular region divided into three units (DL, LEL1, LEL2), Nrx IV with a 6x myc tag (Nrx IV^myc^) and various deletion forms of Nrx IV^myc^ in the N-terminus are represented as Nrx IV^mycΔDL^, Nrx IV^mycΔLEL1^, Nrx IV^mycΔLEL2^, Nrx IV^mycΔDL.LEL1^, Nrx IV^mycΔLEL1.LEL2^, Nrx IV^mycΔNT^ and Nrx IV^mycΔCT^ lacking the cytoplasmic region. (**B–D**) Western blot analysis of wild type (Nrx IV), *arm-GAL4/UAS-nrx IV^myc^* and *arm-GAL4/UAS-nrx IV^myc^*-deletion constructs-expressing embryos using anti-Nrx IV (**B**) antibodies. Note the presence of the endogenous Nrx IV (155 kDa) in all the lanes as well as Nrx IV^myc^ and Nrx IV^myc^ mutant proteins except Nrx IV^mycΔCT^ which is not detected by anti-Nrx IV (**B**, asterisk). Anti-Myc antibodies detect all the Nrx IV^myc^ proteins and not the endogenous Nrx IV (**C**, asterisk) in wild type embryos. In all the lanes, the levels of Cont (**D**) were not affected. Tub was used as protein loading control (**E**).

In the preceding section, we showed that single amino acid changes in the 2^nd^ and 4^th^ laminin G domains affected the function of Nrx IV in the formation of epithelial SJs. We wanted to systematically delete domains in the extracellular region and also the entire cytoplasmic (CT) region in Nrx IV to determine specific contributions of these domains to subcellular localization of Nrx IV and Cont, and the organization of SJs. We divided the Nrx IV extracellular region into three units (i) Discoidin and laminin G domain 1 (DL); (ii) laminin G domain 2-EGF-laminin G domain 3 (LEL1) and (iii) laminin G domain 4-EGF-laminin G domain 5 (LEL2) (Fig. 3A). We generated 6xMyc epitope-tagged full length Nrx IV and deletions corresponding to DL, LEL1, LEL2; and combined deletions (DL-LEL1, LEL1-LEL2), NT (lacking DL-LEL2) and Nrx IV with a transmembrane region but lacking the CT region (Fig. 3A) (*for details refer Materials and Methods*). All *nrx IV^myc^* constructs were placed downstream of *UAS*-sequences to be expressed by tissue specific-GAL4 [41]. Transgenic lines of each of the *nrx IV^myc^* constructs were generated and crossed to *armadillo (arm)*-*GAL4* [38] to confirm the expression and predicted sizes of various Nrx IV^myc^ proteins.

The *GAL4*-driven *nrx IV^myc^* transgenic lines were analyzed by immunoblot analysis (Fig. 3B, C). The wild type (Canton S, lane Nrx IV) served as a control. Since the over-expression of the Nrx IV^myc^ proteins was assessed in the wild type background, immunoblotting against Nrx IV identified the endogenous Nrx IV in all *nrx IV^myc^* lines (Fig. 3B, arrow). All transgenic lines expressed the appropriate Nrx IV-myc-tagged proteins as detected by anti-Nrx IV, although Nrx IV^mycΔCT^ (Fig. 3B, asterisk) could not be detected by anti-Nrx IV antibody raised against the Nrx IV C-terminus [12]. The expression of the Nrx IV-myc-tagged proteins was also confirmed by immunoblotting using anti-Myc antibodies (Fig. 3C), and the levels of Cont in all *nrx IV^myc^* transgenic lines appear equivalent to the wild type embryos (Fig. 3D). Anti-Tubulin levels were used as a loading control (Fig. 3E). Together, the immunoblot analysis using anti-Nrx IV and anti-Myc antibodies confirmed the expression and the expected sizes of the Nrx IV-myc-tagged proteins in respective transgenic lines.

### Laminin G 2-EGF-Laminin G 3 (LEL1) unit of Nrx IV is Essential for Cont Localization

**Figure 4:**
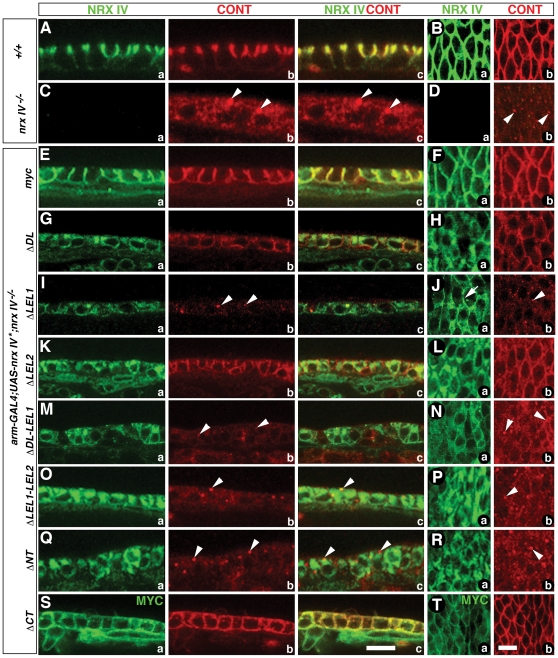
Domain-specific deletions in Nrx IV affect the subcellular localization of Nrx IV and Cont at epithelial SJs. (**A, B**) Wild type epithelia show Nrx IV (**a**) and Cont (**b**) localization at SJs (**a–c**). A surface view of epithelia shows Nrx IV (**Ba**) and Cont (**Bb**) in the epithelial plasma membrane. (**C, D**) *nrx IV^−/−^* shows loss of Nrx IV (**Ca**) and diffuse and punctate localization of Cont (**Cb, Cc**, arrowheads). Epithelial surface view shows absence of Nrx IV (**Da**) and Cont localization in the puncta (**Db**, arrowheads). (**E, F**) Expression of *nrx IV^myc^* in *arm-Gal4/UAS-nrx IV^myc^;nrx IV^−/−^* embryos is able to restore Nrx IV at SJs (**a**) and rescue Cont (**b**) localization at SJs (**a–c**). Surface view reveals a rescue of the membrane localization of Nrx IV (**Fa**) and Cont (**Fb**). (**G, H**) Expression of *nrx IV^mycΔDL^* in *arm-Gal4/UAS-nrx IV^mycΔDL^;nrx IV^−/−^* embryos shows Nrx IV^mycΔDL^ localization mostly in the cytoplasm (**Ga, c, Ha**), while Cont localization, although not enriched at the SJs, is at the membrane (**Gb, c, Hb**). (**I, J**) Deletion of the LEL1 module in *arm-Gal4/UAS-nrx IV^mycΔLEL1^;nrx IV^−/−^* embryos shows localization of Nrx IV in the cytoplasm and to lesser extent in the plasma membrane (**Ia, Ic, Ja**), while Cont fails to localize at the epithelial membrane (**Ib, c, Jb**) and is seen in puncta as observed in *nrx IV* mutants (**Cb,c**). (**K, L**) Nrx IV localization is dramatically reduced in the absence of LEL2 module (**Ka, c, La**), while Cont is targeted to the epithelial membrane and localize at the SJs (**Kb, c, Lb**). (**M–R**) In the absence of DL-LEL1 modules (**M, N**), LEL1-LEL2 modules (**O, P**) and NT (DL-LEL2) modules (**Q, R**) Nrx IV shows cytoplasmic accumulation (**Ma, c, Na; Oa, c, Pa** and **Qa, c, Ra**) and Cont localization is diffuse and in puncta (**Mb, c, Nb; Ob, c, Pb** and **Qb, c, Rb**). (**S, T**) Elimination of the cytoplasmic region of Nrx IV resulted in the targeting of both Nrx IV (**Sa, c, Ta**) and Cont (**Sb, c, Tb**) throughout the epithelial membrane without any specific enrichment at the SJs. Scale bars: 10 µm.

To identify the domains that are responsible for targeting of Nrx IV and Cont to epithelial membrane and SJs, we analyzed the subcellular localization of Myc-tagged Nrx IV deletions in the epidermis of *nrx IV* mutants (Fig. 4). Wild type (*+/+*) embryos show colocalization of Nrx IV (Fig. 4Aa) and Cont (Fig. 4Ab) at the epithelial SJs (Fig. 4Ac, merged image). A surface view of the epithelia from the wild type embryos illustrates the characteristic pattern of membrane localization of Nrx IV (Fig. 4Ba) and Cont (Fig. 4Bb). In *nrx IV^4304^* null mutants (*nrx IV^−/−^*) (Fig. 4Ca) Cont fails to localize to SJs and is instead distributed throughout the cytoplasm and concentrated in small puncta (Fig. 4Cb, Cc, arrowheads) as previously reported [8]. This distribution is also apparent in the surface view of the epithelia (Fig. 4Db, arrowheads). The full length Nrx IV^myc^ is able to restore both Nrx IV (Fig. 4Ea) and Cont (Fig. 4Eb) localization to the epithelial membrane and shows enrichment at the SJs of *nrx IV^−/−^* cells (Fig. 4E and 4F). Interestingly, the transgene lacking the Discoidin-Laminin G 1 in Nrx IV (Nrx IV^mycΔDL^) was partially able to restore Cont localization to the epithelial membrane (Fig. 4Gb), even though it itself failed to localize properly to the membrane (Fig. 4Ga). The surface view of Nrx IV (Fig. 4Ha) and Cont (Fig. 4Hb) show membrane localization of Cont, while Nrx IV^mycΔDL^ is primarily localized in the cytoplasm and shows little, if any localization to the membrane. The transgene lacking the Laminin G 2-EGF-Laminin G 3 domain (Nrx IV^mycΔLEL1^) fails to localize properly to the plasma membrane (Fig. 4Ia), and Cont also is not localized at the plasma membrane or SJs (Fig. 4Ib, arrowhead). Nrx IV membrane localization was observed to some extent in the surface view (Fig. 4Ja, arrow), while Cont is diffuse and accumulates as puncta (Fig. 4Jb, arrowhead). Surprisingly, the *nrx IV* transgene lacking the laminin G 4-EGF-Laminin G 5 (Nrx IV^mycΔLEL2^) failed to localize at the membrane (Fig. 4Ka), but Cont localized to the membrane and in some cells was enriched at the SJs (Fig. 4Kb). Surface view of epithelia show high levels of Nrx IV in the cytoplasm (Fig. 4La) while Cont is targeted to the membrane (Fig. 4Lb). This data underscores the importance of the LEL1 unit of Nrx IV in subcellular localization of Nrx IV and Cont in the epithelia.

Transgene- encoding proteins with larger deletions spanning the Discoidin through LEL1 (Nrx IV^mycΔDL-LEL1^) (Fig. 4M), LEL1 and LEL2 (Nrx IV^mycΔLEL1-LEL2^) (Fig. 4O) and the entire N-terminus except the TM and the CT (Nrx IV^mycΔNT^) (Fig. 4P) (refer Fig. 3A) all failed to localize to SJs or to promote the localization of Cont (Fig. 3Ma, Oa and Qa and Fig. 3Mb, Ob and Qb, respectively). The surface views of Nrx IV (Fig. 4Nd, Pd, Rd) and Cont (Fig. 4Nb, Pb, Rb) show Nrx IV transgenes are mostly in the cytoplasm while Cont has a diffuse and punctate localization (Fig. 4Nb, Pb, Rb, arrowheads). The transgene lacking the C-terminus of Nrx IV (Nrx IV^mycΔCT^) localizes to the plasma membrane, along with Cont, but both the transgene (Fig. 4Sa, 4Ta) and Cont (Fig. 4Sb, 4Tb) do not appear to enrich at the SJs (Fig. 4Sc). Together, these data further indicate that the LEL1 unit of Nrx IV is critical for the restoration of Cont localization at the plasma membrane and that the C-terminus of Nrx IV is essential for restricting the localization of Nrx IV and Cont at the epithelial SJs.

### Biochemical Analysis Reveals Specific Domain Requirement for Nrx IV and Cont Interactions

**Figure 5:**
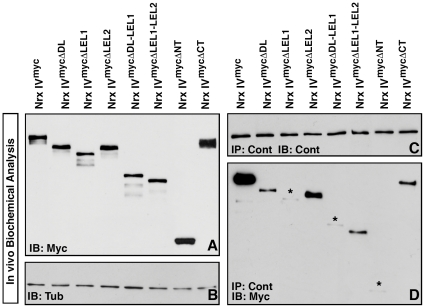
Molecular interactions between Nrx IV and its domain-specific deletion forms with Cont. (**A**) Immunoblot analysis of embryonic lysates from various *nrx IV^myc^* constructs driven by *arm-Gal4* in wild type background show relative protein sizes of the full-length Nrx IV^myc^ and the truncated Nrx IV^myc^ proteins with anti-Myc antibodies. (**B**) Similar levels of protein were loaded in all lanes and are in the same order as in (**A**) and verified by anti-Tub antibodies. (**C**) IP using anti-Cont in all the Nrx IV^myc^ embryos show presence of equal amount of Cont. (**D**) IP using anti-Cont from the various Nrx IV^myc^ embryos reveal a strong association of the Nrx IV^myc^ with Cont. Nrx IV^mycΔLEL2^ also displays an association with Cont that seems stronger than the Nrx IV^mycΔDL^, Nrx IV^mycΔLEL1.LEL2^ and Nrx IV^mycΔCT^ deletions. Nrx IV^mycΔLEL1^, Nrx IV^mycΔDL.LEL1^ and Nrx IV^mycΔNT^ show extremely reduced to almost no association with Cont (asterisks).

We have previously demonstrated that Nrx IV and Cont exist in a molecular complex [8], [13]. The results presented in the preceding section suggest that LEL1 unit is essential for interaction with Cont. To test this hypothesis, we examined the ability of the different Nrx IV transgenes to associate with Cont *in vivo* using IP and immunoblot analyses. Lysates from wild type embryos expressing *nrx IV^myc^* and the *nrx IV^myc^* deletions were immunoblotted with anti-Myc antibodies to confirm the expression of the various myc-tagged transgenes (Fig. 5A). Tubulin was used as loading control (Fig. 5B). These same lysates were then immunoprecipitated with anti-Cont antibodies and probed with anti-cont (Fig. 5C) to confirm the efficacy of the IP, and with anti-myc to determine which transgenes interact with Cont. These assays showed robust interaction between full length Nrx IV^myc^ and Cont (Fig. 5D). The Nrx IV^mycΔLEL2^ protein showed relatively strong interaction with Cont compared to all truncations, although this binding did not reach to similar levels as observed for the full length Nrx IV^myc^ protein. Nrx IV^mycΔDL^, Nrx IV^mycΔLEL1-LEL2^, and Nrx IV^mycΔCT^ showed detectable but significantly reduced binding. Nrx IV^mycΔLEL1^ and Nrx IV^mycΔDL-LEL1^ showed essentially no binding to Cont reaching only the background levels (asterisks, Fig. 5D). Nrx IV^mycΔNT^ showed no association with Cont (Fig. 5D), suggesting that the Cont association with Nrx IV occurs via the extracellular region. Nrx IV^mycΔCT^ retained the ability to associate with Cont although at a reduced level compared to Nrx IV^myc^ full length. These data are consistent with the analysis of the EMS alleles in Fig 1 and the *nrx IV^myc^* deletion analysis described in Fig. 4, and indicate that the domains in the LEL1 unit are essential for Nrx IV and Cont interaction (Fig. 5D).

### Distinct Nrx IV domains regulate the formation and placement of SJs in epithelial cells

**Figure 6:**
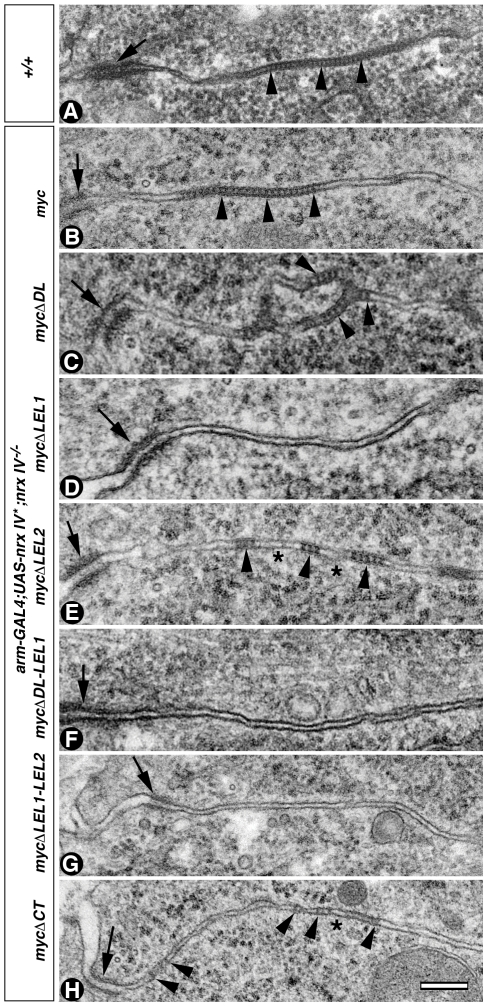
Specific domains in Nrx IV are sufficient to rescue SJ formation in *nrx IV* mutants. (**A**) TEM of wild type embryo shows presence of apical AJs (arrow) followed by distinct ladder-like pleated SJs (arrowheads) between the apicolateral epithelial membranes. (**B**) Epithelial membranes from *arm-Gal4/UAS-nrx IV^myc^*;*nrx IV^−/−^* show rescue of SJs (arrowheads) below AJs (arrow). (**C**) Epithelial membranes from *arm-Gal4/UAS-nrx IV^mycΔDL^*;*nrx IV^−/−^* show formation of SJs between the epithelial membranes (arrowheads) below the AJs (arrow). (**D, F, G**) *arm-Gal4/UAS-nrx IV^mycΔLEL1^*;*nrx IV^−/−^* (**D**), *arm-Gal4/UAS-nrx IV^mycΔDL.LEL1^*;*nrx IV^−/−^* (**F**) and *arm-Gal4/UAS-nrx IV^mycΔLEL1.LEL2^*;*nrx IV^−/−^* (**G**) show absence of SJs between epithelial membranes below the AJs (arrow). (**E**) Embryos lacking the LEL2 module in *arm-Gal4/UAS-nrx IV^mycΔLEL2^*; *nrx IV^−/−^* show presence of patchy SJs between the epithelial cell membranes which are not restricted to the apical SJ domain (arrowheads). Asterisks denote the area missing the SJs. (**H**) Expression of Nrx IV^mycΔCT^ in *arm-Gal4/UAS-nrx IV^mycΔCT^*; *nrx IV^−/−^* shows formation of septa throughout the length of the epithelial membranes (arrows) and not restricted to apicolateral areas. Scale bar: 0.2 µm.

The immunolocalization and biochemical studies identified specific domains in Nrx IV that are required for proper Nrx IV and Cont membrane localization to SJs (Fig. 4) and their ability to associate with Cont (Fig. 5). To investigate how these domains are involved in SJ assembly we carried out TEM analysis of *nrx IV^−/−^* embryos expressing the full length and deletion transgenes outlined in the previous sections. Wild type (*+/+*) embryos showed presence of SJs as distinct ladder-like structures (arrowheads) below the AJs (Fig. 6A, arrows). The loss of SJs observed in *nrx IV* mutants (Fig. 2Aa,b) was completely rescued by full-length Nrx IV^myc^ (Fig. 6B, arrowheads). The SJs in *nrx IV^−/−^* embryos expressing the *nrx IV^myc^* transgene were comparable to those in the wild type embryos, indicating that Nrx IV^myc^ is fully functional and able to rescue SJs in *nrx IV* mutants. TEM analysis of embryos expressing *nrx IV^mycΔDL^* (Fig. 6C) or *nrx IV^mycΔLEL2^* (Fig. 6E) revealed partial rescue of SJs. SJs looked patchy and did not coalesce into apical SJs similar to the EMS mutants. On the other hand, *nrx IV^mycΔLEL1^* (Fig. 6D), *nrx IV^mycΔDL.LEL1^* (Fig. 6F), *nrx IV^mycΔLEL1.LEL2^* (Fig. 6G) and *nrx IV^mycΔNT^* (data not shown) embryos do not show presence of any SJs or isolated septa below the AJs, underlying the importance of the LEL1 unit either alone or in combination for SJ formation. The elimination of the C-terminal region in *arm-Gal4/UAS-nrx IV^mycΔCT^;nrx IV^−/−^* (Fig. 6H) embryos revealed the presence of SJs which were localized more basally to AJs compared to wild type (Fig. 6A) or *nrx IV^myc^* rescued (Fig. 6B) embryos. Together the ultrastructural analyses provide further evidence in favor of the LEL1 module being essential for SJ formation and that the LEL2 module and the C-terminus of Nrx IV are not necessary for septa formation but may be required for proper organization of SJs and their restriction to a more apical region in epithelial cells.

### Full Complement of Transverse Septae at SJs are Required for a Functional Trans-epithelial Barrier

**Figure 7:**
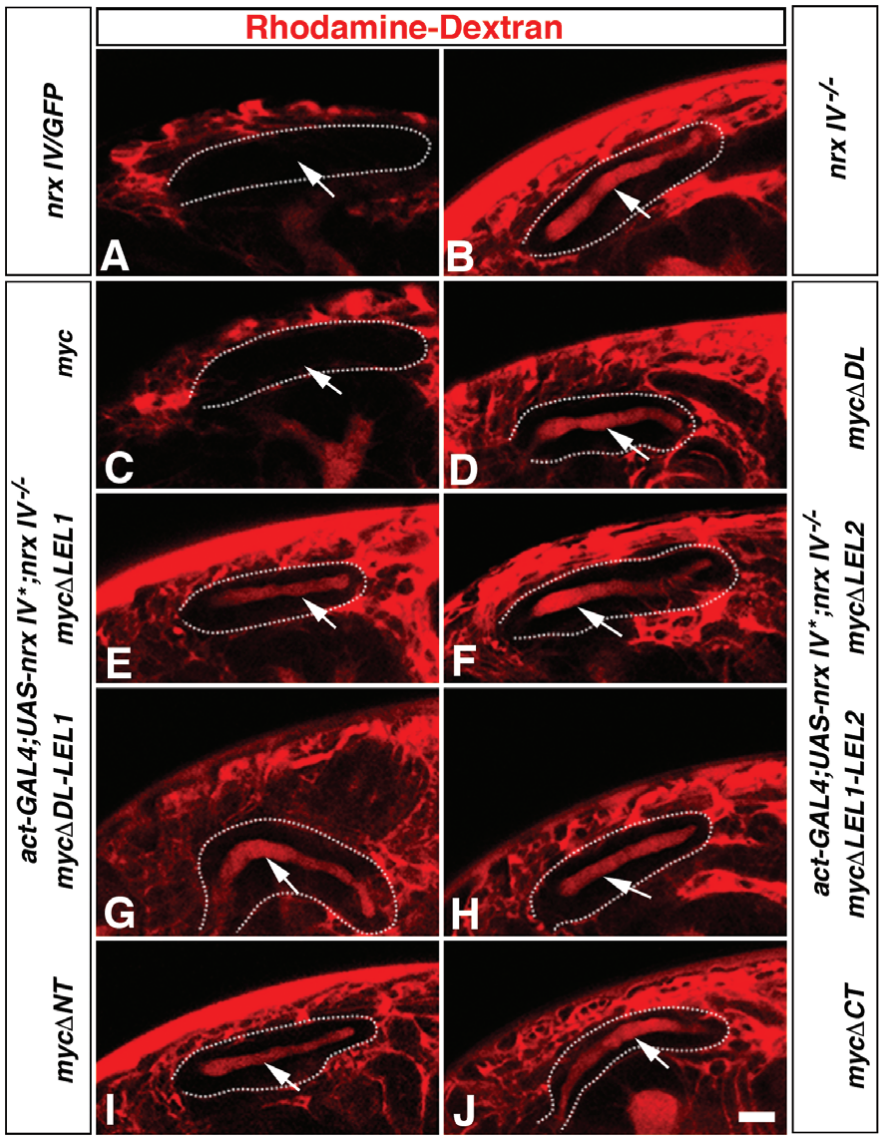
Disruption of the Nrx IV primary structure abrogates its functional role in epithelial barrier formation. (**A, C**) *nrx IV^4304^/GFP* (**A**) and *Act-Gal4/UAS-nrx IV^myc^; nrx IV^−/−^* (**C**) embryos show dye exclusion from the lumen of the salivary gland (arrows). (**B, D–J**) Breakdown of the transepithelial barrier is seen as the lumen of the salivary gland (**B, D–J,** arrow) is filled with the Dextran dye in *nrx IV^−/−^* (**B**), *Act-Gal4/UAS-nrx IV^mycΔDL^; nrx IV^−/−^* (**D**), *Act-Gal4/UAS-nrx IV^mycΔLEL1^; nrx IV^−/−^* (**E**), *Act-Gal4/UAS-nrx IV^mycΔLEL2^; nrx IV^−/−^* (**F**), *Act-Gal4/UAS-nrx IV^mycΔDL-LEL1^; nrx IV^−/−^* (**G**), *Act-Gal4/UAS-nrx IV^mycΔLEL1-LEL2^; nrx IV^−/−^* (**H**), *Act-Gal4/UAS-nrx IV^mycΔNT^; nrx IV^−/−^* (**I**) and *Act-Gal4/UAS-nrx IV^mycΔCT^; nrx IV^−/−^* (**J**) embryos. Scale bar: 50 µm.

The ultrastructural studies in the preceding section revealed phenotypes ranging from a full complement of septae in SJs as seen in the wild type control (Fig. 6A) and the full length *nrx IV^myc^* rescue (Fig. 6B) to complete absence of septa (Fig. 6D, F, G) and intermediate phenotypes with presence of varying degree of septal clusters in the different *nrx IV^myc^* deletions (Fig. 6C, E, H). These observations raised the question of whether the various *nrx IV^myc^* deletion mutants have a functional transepithelial barrier. To address this question, we performed a standard dye exclusion assay [8] in live stage 16–17embryos. We injected a 10 kDa Rhodamine-Dextran dye into the posterior body cavity of live embryos (Fig. 7, refer *Materials and Methods*) to assess the ability of the salivary gland epithelia to exclude the dye. After the dye injection, confocal sections were acquired at 15 minutes post injection and quantified (n = 15) for presence of intact barrier. For the expression of various *nrx IV^myc^* transgenes in *nrx IV^−/−^* mutant background, we utilized a stronger and ubiquitous *Act5C-Gal4* driver. In control heterozygous *nrx IV^4304^/GFP* embryos the dye remained excluded from salivary glands of all embryos (Fig. 7A, arrow) while *nrx IV^−/−^* mutants failed to exclude the dye, which breached into the lumen of the salivary glands within 10 minutes after injection (Fig. 7B). While a statistically significant rescue of dye exclusion was observed in full length *nrx IV^myc^* transgene (Fig. 7C, arrow) compared to *nrx IV* mutants (Fig. 7B, arrow), none of the *nrx IV^myc^* deletion constructs (Fig. 7D–J, arrow) were able to exclude the dye from the lumen of the salivary gland (Figure S1). These data suggest that a full complement of SJs is required to establish a functional paracellular barrier in the epithelial cells.

### LEL1 domain of Nrx IV is Sufficient for Nrx IV and Cont Interactions and Localization to the Apico-Lateral Epithelial Membrane

**Figure 8:**
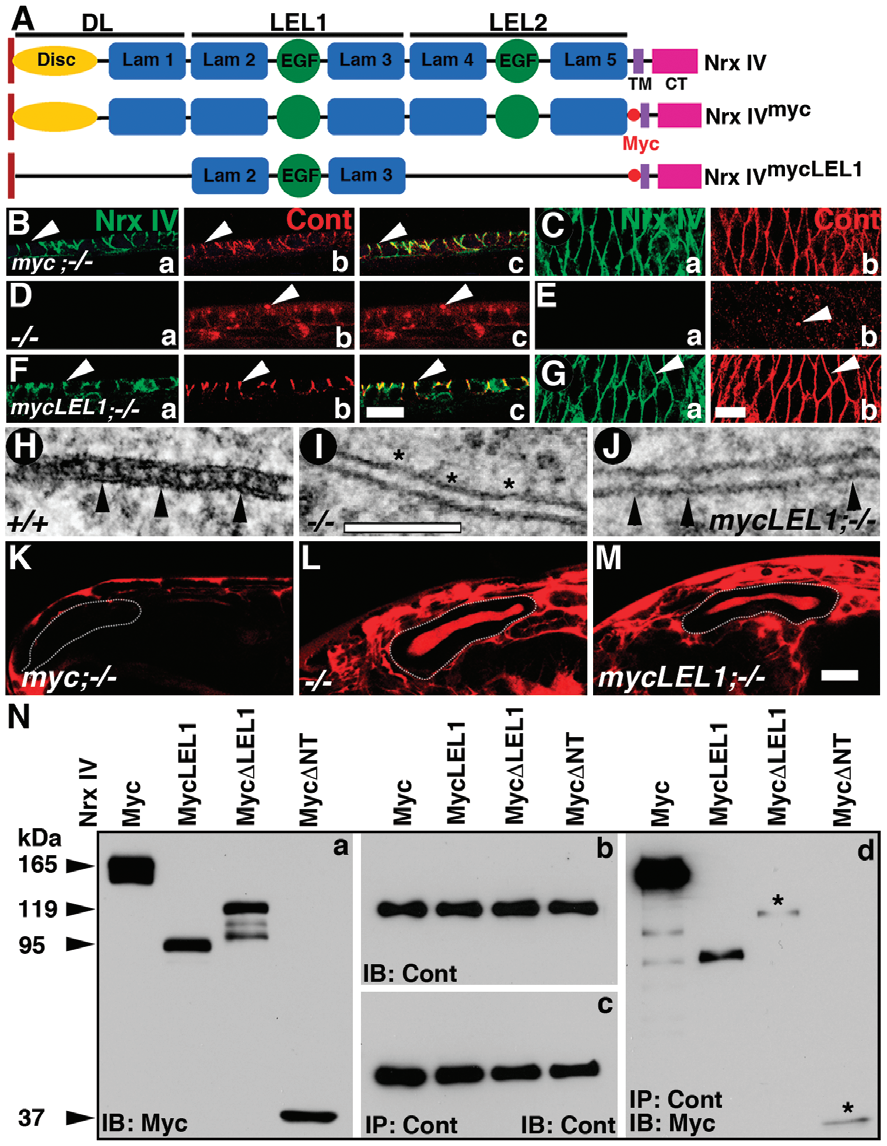
The LEL1 module of Nrx IV is sufficient for Nrx IV and Cont interactions and apico-lateral membrane localization. (**A**) Schematic representation of Nrx IV^mycLEL1^ polypeptide with respect to Nrx IV and Nrx IV^myc^ structures. (**B, C**) In *arm-Gal4/UAS-Nrx IV^myc^;nrx IV^−/−^* embryos Nrx IV (**Ba, Bc**) and Cont (**Bb, Bc**) localize to the epithelial membrane and show enrichment at the apico-lateral region where SJs are formed (arrowheads). Surface view of epithelia also shows a sharp membrane localization of Nrx IV (**Ca**) and Cont (**Cb**). (**D, E**) *nrx IV^−/−^* embryos show loss of Nrx IV (**Da, Ea**) and a diffuse and punctate distribution of Cont (**Db, Dc, Eb**, arrowhead). (**F, G**) *arm-Gal4/UAS-nrx IV^mycLEL1^; nrx IV^−/−^* show localization of Nrx IV (Fa, Fc, Ga) and Cont (**Fb, Fc, Gb**) in plasma membrane (arrowheads) and restriction to the apico-lateral region. (**H–J**) TEM of epithelia show presence of septa in wild type (**H**, arrowheads), a complete lack of septa in *nrx IV^−/−^* embryos (**I**, asterisks) and presence of few isolated septae in *arm-Gal4/UAS-nrx IV^mycLEL1^; nrx IV^−/−^* embryos (**J**, arrowheads). (**K–M**) Injected Dextran dye in the posterior body cavity of embryos fail to penetrate the salivary glands in presence of functional SJs in wild type (**K**) while the dye penetrates freely in *nrx IV^−/−^* embryos (**L**) and *Act-Gal4/UAS-nrx IV^mycLEL1^; nrx IV^−/−^* (**M**) embryos. (**N**) Western blots of embryonic lysates from *arm-Gal4/UAS-nrx IV^myc^*, *arm-Gal4/UAS-nrx IV^mycLEL1^*, *arm-Gal4/UAS-nrx IV^mycΔLEL1^* and *arm-Gal4/UAS-nrx IV^mycΔNT^* immunoblotted with anti-Myc antibodies shows an ∼92 kDa band in *arm-Gal4/UAS-nrx IV^mycLEL1^*. The levels of Cont in all genotypes are also shown (**b**). Embryonic lysates from all genotypes were immunoprecipitated using anti-Cont antibodies and immunoblotted with anti-Cont antibodies (**c**) and anti-Myc antibodies (**d**). Nrx IV^mycLEL1^ is capable of associating with Cont above the background levels seen in Nrx IV^mycΔLEL1^ and Nrx IV^mycΔNT^ lanes (**d**, asterisks). Scale bars: B–G, 10 µm; H–J, 0.1 µm; K–M, 50 µm.

Multiple lines of evidence throughout the course of this study highlighted the importance of the LEL1 module of Nrx IV in proper epithelial Cont localization and complex formation. In order to determine whether the LEL1 domain is sufficient for proper epithelial localization of both Nrx IV and Cont, formation of functional SJs and biochemical interaction with Cont, we generated a transgenic fly line with only the LEL1 module of the ECD of Nrx IV tagged with 6X Myc together with the TM and CT domains (Fig. 8A) and termed it *nrx IV^mycLEL1^*.

We first studied the localization of Nrx IV and Cont in *arm-Gal4/UAS- nrx IV^mycLEL1^; nrx IV^−/−^* (Fig. 8F, G) and compared it with *arm-Gal4/UAS- nrx IV^myc^; nrx IV^−/−^* (Fig. 8B, C) and *nrx IV^−/−^*(Fig. 8D, E) embryos. To our surprise, the Nrx IV^mycLEL1^ was well localized to plasma membrane (Fig. 8Fa, Ga, arrowheads) and restored localization of Cont (Fig. 8Fb, Gb, arrowheads). Both showed enrichment at the apico-lateral region where SJs are established. This localization is similar to that seen with the *nrx IV^myc^* transgene (Fig. 8B, arrowheads). *nrx IV* mutants, on the other hand, failed to localize Cont to the epithelial membrane (Fig. 8Db, c, Eb). These studies clearly identify the extracellular LEL1 module of Nrx IV to be sufficient for apico-lateral localization of Nrx IV and Cont.

We next wanted to know whether the LEL1 domain is capable of organizing functional SJs. To address this question, we performed TEM of epithelia (Fig. 8H–J) and dye exclusion assay (Fig. 8K–M) on wild type (Fig. 8H, K), *nrx IV^−/−^* (Fig. 8I, L) and *nrx IV^mycLEL1^* (Fig. 8J, M) embryos. The TEM analysis showed the presence of SJs in the control wild type (Fig. 8H) and an intact functional barrier (Fig. 8K) while *nrx IV^−/−^* showed absence of SJs (Fig. 8I) and dye penetration in the lumen of the salivary gland (Fig. 8L). TEM of *nrx IV^mycLEL1^* showed a failure to form characteristic SJs (Fig. 8J), but showed presence of occasional septae (Fig. 8J, arrowheads) and electron densities between the epithelial membranes indicating that the LEL1 module alone may not be sufficient to establish a full complement of SJs normally observed in the wild type epithelia. Consistent with this observation, *nrx IV^mycLEL1^* embryos were unable to form a fully functional barrier to exclude the dye from the salivary glands (Fig. 8M) compared to *nrx IV^myc^* embryos (Fig. 8K).

Finally, we assessed the ability of LEL1 domain to interact with Cont by IP/immunoblot analysis in wild type embryos. We confirmed the size of the Nrx IV^mycLEL1^ construct to be ∼92 kDa by immunoblotting embryonic lysate with anti-Myc antibody (Fig. 8Na) with respect to Nrx IV^myc^, Nrx IV^mycΔLEL1^ and Nrx IV^mycΔNT^. The levels of Cont were similar (Fig. 8Nb) in all four genotypes. Upon IP with anti-Cont, the lysates were probed for Cont (Fig. 8Nc) to confirm the efficacy of the IPs and with anti-Myc to determine if *nrx IV^mycLEL1^* transgene retained its ability to interact with Cont. *nrx IV^mycLEL1^* was capable of associating with Cont, although at a lower level compared to *nrx IV^myc^* (Fig. 8Nd). The *nrx IV^mycΔLEL1^* and *nrx IV^mycΔNT^* showed essentially background levels as observed previously (Fig. 5D). These studies show that the LEL1 module of Nrx IV is sufficient to localize Nrx IV and Cont to the apico-lateral area of the epithelial plasma membrane and its ability, at least in part, to associate with Cont in a biochemical complex.

## Discussion

The objective of the current study was to dissect the mechanism of SJ assembly, specifically by addressing how conserved domains within Nrx IV contribute to the localization, interaction and assembly of SJ proteins. The data suggest that there are at least two steps in the assembly of these proteins into SJs–trafficking to the plasma membrane and subsequent organization into apical septae. The incorporation of Nrx IV into the plasma membrane requires most, if not all, of the extracellular domains of Nrx IV, but does not require the C-terminal cytoplasmic domain. The N-terminal LEL1 module is particularly important for plasma membrane localization, since a single point mutation in the second Laminin G domain dramatically reduces membrane accumulation. The membrane localization of the Nrx IV-binding partner Cont does not appear to require Nrx IV localization to the membrane, as mutations that disrupt Nrx IV localization do not necessarily have a detrimental effect on Cont localization to the plasma membrane. It is possible, though, that the membrane accumulation of Cont does require binding to Nrx IV at some point during trafficking to membrane, since interactions between the two proteins in IP assays correlate with membrane localization. Interestingly, this localization of Nrx IV (and Cont) to the membrane appears to be sufficient for the formation of small isolated septa observed by TEM, although these septae are usually distributed along the entire lateral surface and are incapable of establishing a functional paracellular barrier.

### Laminin G domains in Nrx IV and their role in SJ formation

Accumulated genetic and molecular evidence has linked Nrx IV with Cont and Nrg into an adhesive mechanism which mediates formation of SJs to ensure the formation and maintenance of a paracellular barrier in epithelial and neuronal cells [2], [8], [14]. While the exact molecular underpinnings of SJ formation and barrier function are still poorly understood, key molecular information about specific domains in Nrx IV suggests that individual domains play unique roles in the function of Nrx IV (refer Table 1). The 5 laminin G domains form the major portion of the extracellular region of Nrx IV protein (Fig. 1). The laminin G domains were originally identified as fivefold repetition of about 158 to 180 residues in the C-terminal globular domain of lamininα-1 chain [42], but are found in a multitude of diverse proteins which have roles in cell adhesion, signaling, migration, assembly and differentiation [43], [44]. Sequence analysis of the hypomorphic mutations in *nrx IV* indicate that within the laminin G domains single amino acid changes lead to functional deficits in Nrx IV, such as those observed in *nrx IV^711^* allele. Here Nrx IV is expressed almost at normal levels and does not completely lose its ability to form septa but fails to establish a well-defined series of transverse septae characteristic of SJs as seen in the wild type cells. A similar effect is observed in an allele that has dramatically reduced level of Nrx IV (e.g. *nrx IV^2511^*) which leads to reduction in the number of septa at the SJs. These observations allow us to conclude that Nrx IV levels in the epithelia are important to form linked arrays of septae, and that individual laminin G domains may also have specific roles in organizing septae into contiguous circumferential ribbons characteristic of SJs.

**Table 1:** Summary of the protein localization, biochemical interactions and barrier formation abilities of Nrx IV and its various mutant forms.

Genotype	Membrane Localization		SJ Localization		Apical SJ Formation	Lateral Septae Formation	Binding with Cont	Barrier Function
	Nrx IV	Cont	Nrx IV	Cont				
*nrx IV^+/+^*	+++	+++	+++	+++	+++	-	+++	+++^c^
*nrx IV^−/−^*	-	a	-	-	NA	NA	NA	-
*nrx IV^myc^* ^***b***^	+++	+++	++	++	+++	-	+++	++
*nrx IV^mycΔDL^*	+	++	-	-	++	+	+	-
*nrx IV^mycΔLEL1^*	+	a	-	-	-	-	-	-
*nrx IV^mycΔLEL2^*	+	++	-	++	++	+	++	-
*nrx IV^mycΔDL-LEL1^*	-	a	-	-	-	-	-	-
*nrx IV^mycΔLEL1-LEL2^*	-	a	-	-	-	-	+	-
*nrx IV^mycΔNT^*	-	a	-	-	-	-	-	-
*nrx IV^mycΔCT^*	+++	+++	-	-	++	+	+	-
*nrx IV^mycLEL1^*	++	++	+	+	+	+	+	-
*nrx IV^319^*	++	++	++	++	++	-	NA	NA
*nrx IV^711^*	+	++	+	++	++	+	NA	NA
*nrx IV^2511^*	+	+	+	+	+	-	NA	NA

++ and + are comparisons against wild type which is represented as +++.

-Represents a phenotype that is observed in *nrx IV* null alleles.

a Represents punctate localization of Cont in the cytoplasm.

b The genotype of all *nrx IV^myc^* and *nrx IV^myc^*-deletion lines is (arm-GAL4/UAS-nrx IV^myc*^;nrx IV^−/−^).

c The genotype of all *nrx IV^myc^* and *nrx IV^myc^*-deletion lines used for dye injections is (act-GAL4/UAS-nrx IV^myc*^;nrx IV^−/−^).

NA-Not applicable

Our systematic deletion analysis indicates that the laminin G 2-EGF-laminin G 3 (LEL1) unit of Nrx IV is essential for several functions of Nrx IV. Nrx IV construct lacking LEL1 (Nrx IV^mycΔLEL1^) are primarily cytoplasmic although surface views show weak localization at the epithelial membrane. They also failed to interact with Cont in *in vivo* IP assays or to rescue Cont localization and SJ formation. On the other hand, Nrx IV^mycΔDL^ and Nrx IV^mycΔLEL2^ proteins localized poorly to the membrane but both bound to Cont, rescued Cont membrane localization and promoted the formation of some limited septae. Thus binding to Cont is an important aspect for SJ formation. The failure of the EMS allele *nrx IV^711^* to support SJ formation and Cont localization suggests that the second laminin G domain of LEL1 is critical to Nrx IV function, but does not allow us to rule out a role for other motifs in LEL1 (Laminin G domain 3 and/or the EGF domain) (Table 1). Finally, the expression of the LEL1 module alone of the ECD of Nrx IV in *nrx IV^−/−^* could rescue the membrane localizations of Nrx IV and Cont as well as their in vivo biochemical complex formation. However, a lack of full complement of SJs, as seen in these mutants by TEM, presents an interesting caveat. While, on one hand, LEL1 module is sufficient for the membrane localization of Nrx IV and Cont, it could not rescue SJs as is seen in the wild type epithelia, suggesting that the rest of the domains in Nrx IV are likely to be also required for interacting with other known and yet unknown SJ proteins for the proper assembly of the SJs and the creation of an intact paracellular barrier. It is also possible that lack of a particular domain or module may impart additional functional characteristics to the remaining protein which could lead to new intermolecular interactions. While this remains a possibility, we cannot experimentally rule out such functions or their contributions in the observed phenotypes.

### Nrx IV Domains and Molecular Interactions at the SJs

SJ assembly is a complex process mediated by multiple polypeptides that form both homo and heterotypic interactions. Previous studies have established that Nrx IV, Cont and Nrg are required for formation of SJs [8], [12], [13], [26]. Our observations, as reported here, indicate that the LEL1 unit of Nrx IV is critically required for Nrx IV interaction with Cont. However, Nrx IV transgenes lacking the DL, LEL2 or CT domains also failed to completely restore SJ formation in *nrx IV* mutant embryos (Table 1) suggesting that these domains are also required for some critical function, most likely by mediating interactions with other SJ proteins or by promoting adhesion or trafficking to cell membranes as has been reported for Caspr and Cont [33], [34]. For example, the C-terminal domain of Nrx IV appears to be necessary for binding to the cytoskeletal linker Cora. Cora, like Nrx IV, is required for SJ assembly [12], [17], suggesting that the interaction between these two proteins is critical for SJ assembly. Consistent with this hypothesis, we observed a severe reduction in the number of septa in *nrx IV^mycΔCT^* embryos and a redistribution of the septa along the apico-basal axis. These observations confirm that Cora is a critical regulator of Nrx IV function. Interestingly, deletion of the cytoplasmic region of Nrx IV also led to reduced interaction with the GPI linked extracellular ligand Cont, suggesting that the interaction of Nrx IV with cytoplasmic Cora may stabilize the Nrx IV/Cont complex at the membrane. Thus, the formation of SJs depends on multiple players, most likely interacting at different steps during the assembly of SJs. Our results highlight the complexity of SJ formation, and suggest that the assembly and/or stability of this complex is dependent on multiple intermolecular interactions between SJ proteins and the cytoskeleton.

Given the structural and functional similarities between the invertebrate and vertebrate SJs [3], our studies may provide clues about the specific domain functions of vertebrate Caspr that underlie the formation of axo-glial SJs. Deletion of similar units in Caspr may provide fundamental insights into how the protein-protein interactions occur at the paranodes of myelinated axons and how the axo-glial SJs apparatus is assembled, which are central to the organization and maintenance of axonal domains required for saltatory nerve conduction.

## Supporting Information

Figure S1 **Disruption in the primary structure of Nrx IV abolishes barrier formation.** Rhodamine-dextran dye injected in stage 16 embryos of *nrx IV/twi-GFP* show an intact paracellular barrier. This barrier is compromised in *nrx IV^−/−^* embryos. Expression of Nrx IV from *Act-Gal4/UAS-nrx IV^myc^* in *nrx IV^−/−^* embryos significantly restores the paracellular barrier. *nrx IV^−/−^* embryos carrying transgenes expressing various domain deletion forms of Nrx IV are unable to create a functional barrier and thus fail to exclude the injected dye from the salivary glands.
(TIF)
