# Supplementary figures and images for: A Laminin G-EGF-Laminin G Module in Neurexin IV Is Essential for the Apico-Lateral Localization of Contactin and Organization of Septate Junctions
Source: PLoS One. 2011 Oct 14;6(10):e25926. doi: 10.1371/journal.pone.0025926 (PMC3195077; doi:10.1371/journal.pone.0025926)

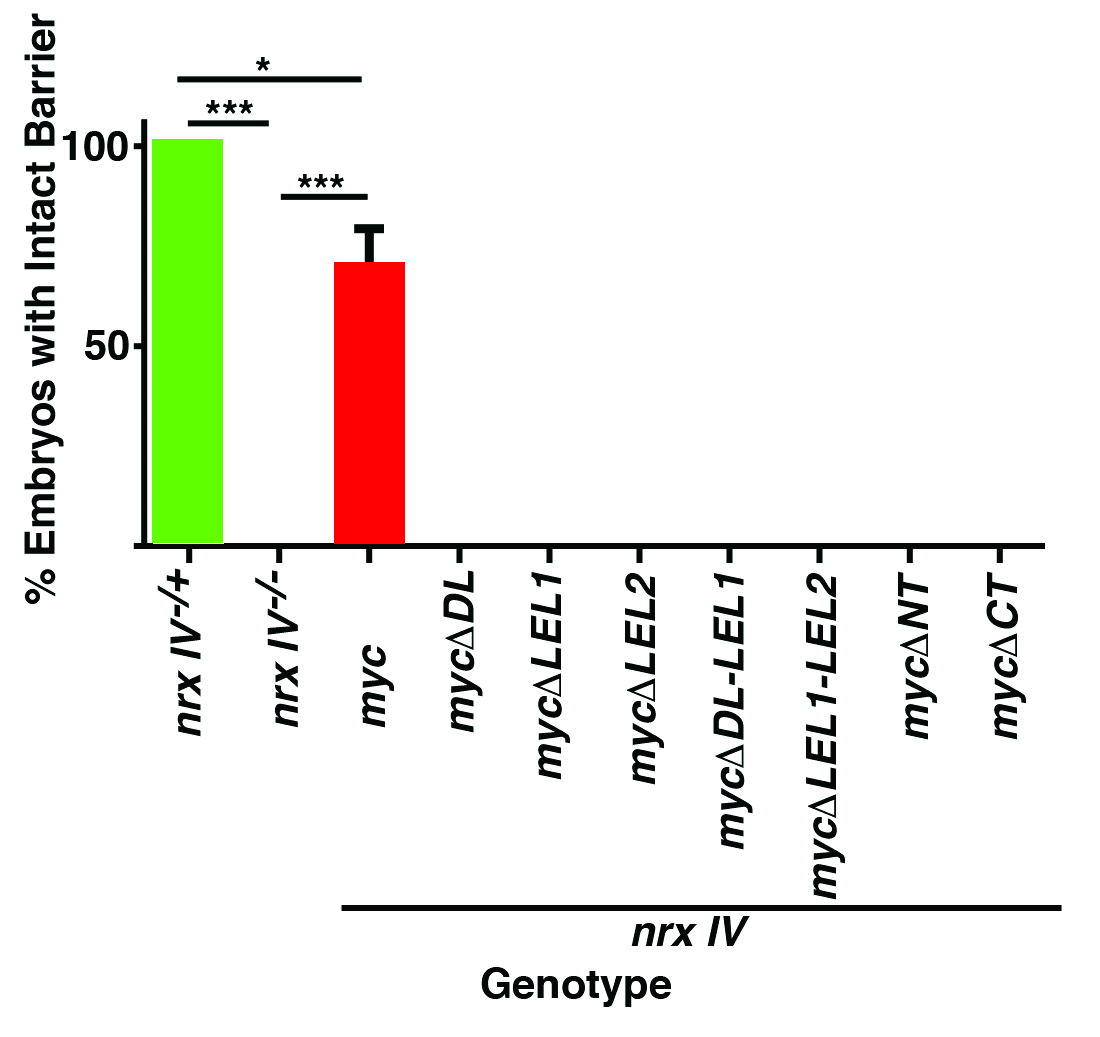

Supplement: Figure S1 — Disruption in the primary structure of Nrx IV abolishes barrier formation. Rhodamine-dextran dye injected in stage 16 embryos of nrx IV/twi-GFP show an intact paracellular barrier. This barrier is compromised in nrx IV−/− embryos. Expression of Nrx IV from Act-Gal4/UAS-nrx IVmyc in nrx IV−/− embryos significantly restores the paracellular barrier. nrx IV−/− embryos carrying transgenes expressing various domain deletion forms of Nrx IV are unable to create a functional barrier and thus fail to exclude the injected dye from the salivary glands. (TIF) [file pone.0025926.s001.tif]
